# Vonoprazan on the Eradication of *Helicobacter pylori* Infection

**DOI:** 10.5152/tjg.2022.211041

**Published:** 2023-03-01

**Authors:** Jiaming Huang, Ye Lin

**Affiliations:** Department of Gastroenterology, Ganzhou People’s Hospital, Jiangxi, China

**Keywords:** *Adverse effects*, eradication rate, Helicobacter pylori, quadruple therapy, vonoprazan

## Abstract

**Background:**

This study aimed to investigate the efficacy and safety of vonoprazan in the eradication of *Helicobacter pylori* (H. pylori).

**Methods:**

A total of 120 cases of *H. pylori*-infected outpatients were selected and randomly divided into the traditional quadruple therapy, vonoprazan triple therapy, and vonoprazan quadruple therapy groups. The traditional quadruple therapy group patients were orally treated with esomeprazole (20 mg) 30 minutes before breakfast and supper, amoxicillin (1000 mg orally) 30 minutes after breakfast and supper, furazolidone (100 mg orally) 30 minutes after breakfast and supper, and bismuth potassium citrate (0.6 g orally) 30 minutes before breakfast and supper. The vonoprazan triple therapy group patients were treated with vonoprazan (20 mg orally) 30 minutes following breakfast and supper, amoxicillin (1000 mg orally) 30 minutes following breakfast and supper, and bismuth potassium citrate (0.6 g orally) 30 minutes before breakfast and supper. The vonoprazan quadruple therapy group patients were treated with vonoprazan (20 mg orally) 30 minutes following breakfast and supper, amoxicillin (1000 mg orally) 30 minutes after breakfast and supper, furazolidone (100 mg orally) 30 minutes after breakfast and supper, and bismuth potassium citrate (0.6 g orally) 30 minutes before breakfast and supper. The 3 groups were treated for 14 days, and adverse reactions, such as vomiting and abdominal distension, were recorded during the treatment period. The ^14^C urea breath test was used to detect whether *H. pylori* was successfully eradicated in the patients.

**Results:**

The eradication rates of the vonoprazan triple therapy, vonoprazan quadruple therapy, and the traditional quadruple therapy groups were 80%, 95%, and 97.5%, respectively. The eradication rate was higher in the vonoprazan triple therapy and in the vonoprazan quadruple therapy groups compared with that noted in the control group. The adverse reactions were mild in these groups, and the main adverse reactions were nausea, abdominal distension, diarrhea, and constipation. The adverse reaction rate was 25%, 7.5%, and 15%, respectively. This rate was lower in the vonoprazan triple therapy and vonoprazan quadruple therapy groups than that noted in the control group.

**Conclusion:**

Both vonoprazan triple therapy and vonoprazan quadruple therapy regimens could increase the eradication rate of *H. pylori*. Vonoprazan triple therapy exhibited reduced side effects and could be applied in the eradication of *H. pylori* in the clinic.

**Main Points**

- A total of 120 cases of H. pylori-infected outpatients were selected and randomly divided into the traditional quadruple therapy, vonoprazan triple therapy, and vonoprazan quadruple therapy groups.
- Both vonoprazan triple therapy and vonoprazan quadruple therapy regimens could increase the eradication rate of H. pylori.
- Vonoprazan triple therapy exhibited reduced side effects and could be applied in the eradication of H. pylori in the clinic.

## Introduction

*Helicobacter* *pylori* (*H. pylori*) is a spiral micro-aerophilic gram-negative bacterium. It is not only the common cause of chronic gastritis but also a pathogenic factor of peptic ulcer, atrophic gastritis, gastric cancer, and gastric mucosa-associated lymphoid tissue lymphoma.^1-4^ It can be easily transmitted through fecal–oral and oral–oral routes or by contact with saliva from infected individuals and by contaminated food. Therefore, the infection rate in the population is very high.^5-6^ In China, the infection rate is as high as 56%. Kyoto Global Consensus recommends eradication treatment for these patients^6^ and drug treatment is the only method used to eradicate *H. pylori* infection.

Drug resistance and CYP2C19 gene polymorphisms noted in host organisms reduce the eradication rate of *H. pylori*. Specifically, the rate is lower than 80% following conventional triple treatment with proton pump inhibitor (PPI), amoxicillin, and furazolidone.^7-13^ The eradication rate can only be increased by 5% by prolonging the treatment to 10 days. The recommended use of antibiotics results in antibiotic resistance of *H. pylori*. Specifically, the resistance rate range of *H. pylori* for metronidazole is 60%-70%, for clarithromycin 20%-38%, and for levofloxacin 30%-38%.^14^ The treatment with high resistance rate drugs can significantly reduce the eradication rate.^15,16^ Quadruple treatment with bismuth is recommended as the first-line treatment.^17^ However, as the resistance of *H. pylori* to these drugs increases, the eradication rate caused by quadruple treatment methods is decreased.^17-21^ In certain patients, treatment is halted due to adverse effects. Therefore, it is imperative to identify a solution that can increase the eradication rate of *H. pylori* and reduce the adverse effects of the treatment.

Vonoprazan is a new proton pump inhibitor, which inhibits the binding of K^+^ ions and stops the exchange of H^+^-K^+^. It exerts sustained inhibition of acid secretion.^22-25^ Vonoprazan can inhibit both the activated and resilient proton pump, which can achieve stronger and longer-lasting suppression of gastric acid secretion than PPIs.^23,26^ This compound was approved for market sale in Japan in 2015 and it was recommended as a first-line treatment for *H. pylori* eradication by the Japanese *H. pylori* infection treatment guidelines in 2016. However, certain effects of the combination of vonoprazan plus bismuth triple therapy and vonoprazan plus bismuth quadruple therapy have not been previously investigated in the treatment of *H. pylori* eradication. Therefore, we discussed the effects of vonoprazan plus bismuth triple therapy and vonoprazan plus bismuth quadruple therapy in the eradication of *H. pylori* infection and the associated adverse effects.

## Materials and Methods

The present study was approved by Ganzhou People’s Hospital Ethics Committee with approval number TY-ZKY2011-011-01.

A total of 120 *H. pylori*-positive patients who agreed to *H. pylori* eradication treatment were selected in our outpatient department between July and November 2021. The inclusion criteria used were as follows: (1) age between 18 and 60, with no restrictions on the gender; (2) confirmation of *H. pylori* infection by the ^14^C urea breath test; (3) patients who did not receive PPI, bismuth, vonoprazan, and antibiotics for the last month; and (4) patients who agreed to participate in the research and signed the informed consent form. The following exclusion criteria were used: (1) patients who had significant hepatic or renal disease; (2) patients who had taken PPI, bismuth, vonoprazan, and antibiotics in the last month; (3) patients who were allergic to penicillin, PPI, bismuth or vonoprazan; and (4) patients who did not accept to participate in the follow-up periods as required.

### Treatment

A total of 120 *H. pylori*-infected patients were randomly divided into the traditional quadruple therapy, the vonoprazan triple therapy, and the vonoprazan quadruple therapy groups. Each group contained 40 patients.

The traditional quadruple therapy group patients were provided with esomeprazole (AstraZenaca Pharmaceutical Corporation London UK) administered at 20 mg orally 30 minutes before breakfast and supper, amoxicillin (Federal drug Corporation Santa Barbara US) administered at 1000 mg orally 30 minutes after breakfast and supper, furazolidone (LiuYe biotechnology Corporation Shandong China) administered at 100 mg orally 30 minutes after breakfast and supper, and bismuth potassium citrate (LiZhu Pharmaceutical Corporation Jiangsu China) administered at 0.6 g orally 30 minutes before breakfast and supper.

The patients in the vonoprazan triple therapy group were administered with vonoprazan (20 mg orally) 30 minutes after breakfast and supper, amoxicillin (1000 mg orally) 30 minutes after breakfast and supper, and bismuth potassium citrate (0.6 g orally) 30 minutes before breakfast and supper.

The patients of the vonoprazan quadruple therapy group were orally administered with 20 mg vonoprazan, 1000 mg amoxicillin, and 100 mg furazolidone 30 minutes after breakfast and supper, while they were also orally administered with 0.6 g bismuth potassium citrate orally 30 minutes before breakfast and supper.

All patients were treated for 14 days and subsequently were followed up for an additional 4 weeks. They were assessed with the ^14^C urea breath test following 1 month of drug withdrawal. The treatment duration and antimicrobial dosages were determined according to the *H. pylori* guidelines published in 2019.

### Observation Indicators

These were divided into main and secondary indicators for observation. The main indicator used was the *H. pylori* eradication rate, whereas the negative ^14^C urea breath test was considered a successful eradication of *H. pylori*. The secondary indicator included the adverse effect rate, such as nausea, vomiting, diarrhea, abdominal distention, and constipation.

### Statistical Analysis

Statistical Package for the Social Sciences 20.0 software (IBM Corp.; Armonk, NY, USA) was used for statistical analysis. The chi-square test was conducted to measure the *H. pylori* eradication rate and the adverse effect rate. *P*  < .05 was considered to indicate a significant difference.

## Results

### Baseline Characteristics

All patients were continuously treated for 14 days. The patients were followed up and accepted the ^14^C urea breath test after 1 month of drug withdrawal. No significant differences were noted in the gender, age, height, and body mass among the 3 groups of patients (Table 1).

### Eradication of ***H. pylori*** Infection

**Figure 1.**
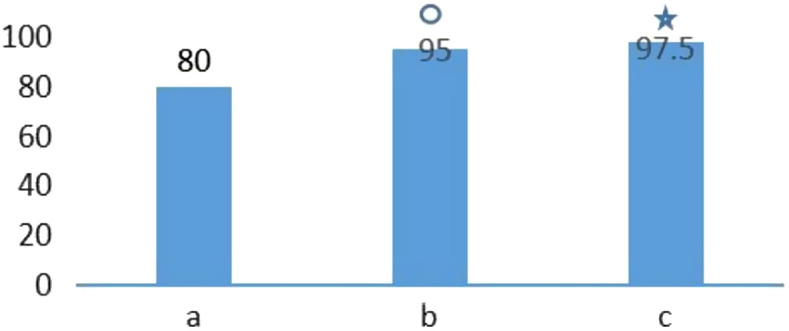
Comparison of the *H. pylori* eradication rate of the 3 groups. (A) Traditional quadruple therapy group; (B) vonoprazan triple therapy group; (C) vonoprazan quadruple therapy group. Compared with the traditional quadruple therapy group *P*  < .05; 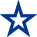 compared with the traditional quadruple therapy group *P*  <.05.

All patients accepted the ^14^C urea breath test after 1 month of drug withdrawal. The eradication rate among the 3 groups was 95%, 97.5%, and 80.0%, respectively, while the eradication rate was significantly higher in both the vonoprazan triple therapy and the vonoprazan quadruple therapy groups than that noted in the control group (Table 2 and Figure 1).

### Adverse Effects

**Figure 2.**
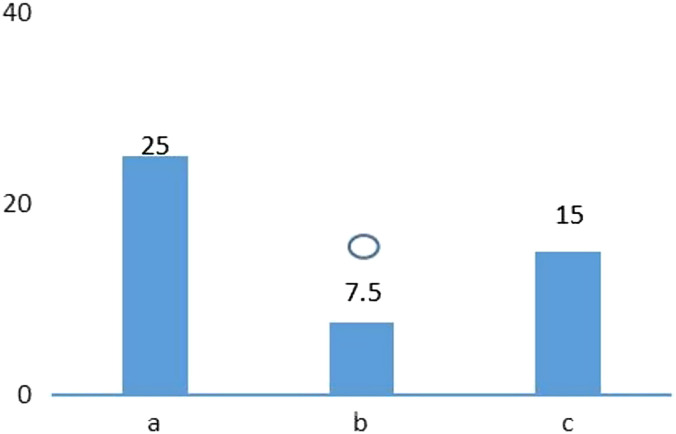
Comparison of adverse effects of the 3 groups. (A) Traditional quadruple therapy group; (B) vonoprazan triple therapy group; (C) vonoprazan quadruple therapy group. 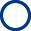 Compared with traditional quadruple therapy group *P*  < .05.

Specific adverse effects were observed in the 3 groups, such as nausea, vomiting, diarrhea, abdominal distention, and constipation. The adverse effect rates noted for the 3 groups were 25%, 7.5%, and 15%, respectively. The adverse effect rate was significantly lower in the vonoprazan triple therapy group than that of the control group (Table 3 and Figure 2).

Comparison between vonoprazan triple therapy and vonoprazan quadruple therapy groups.

Both groups exhibited a high eradication rate, and no significant differences were noted in the eradication rate of the vonoprazan triple therapy and vonoprazan quadruple therapy groups. The adverse effect rate was slightly lower in the vonoprazan triple therapy group than that noted in the vonoprazan quadruple therapy group. However, no significant differences were noted.

## Discussion

*H.* *pylori* is a gram-negative bacterium, which resides in the stomach and duodenum. The infection rate of *H. pylori* is very high, notably in developing countries. In China, the infection rate in adults is 58.07%.^27,28^ *H. pylori* infection is not only the cause of peptic ulcers but it is also associated with atrophic gastritis, gastric cancer, and gastro-esophageal reflux disease.^7,29-31^ Due to its high infection rate and close association with the development of digestive diseases, it is considered a research hotspot.

According to the fifth national guideline for *H. pylori* infection, peptic ulcer and mucosa associated lymphoid tissue (MALT) lymphoma are the indicators of *H. pylori* eradication. The eradication of this microorganism is the first line of prevention of gastric cancer.^32^ The guideline recommends conventional quadruple therapy as the empirical way for the eradication of *H. pylori*. In the present study, conventional quadruple therapy included the following compounds and doses: esomeprazole (20 mg twice a day) before a meal, bismuth potassium citrate (0.6 g twice a day) before a meal, amoxicillin (1.0 g twice a day) after a meal, and furazolidone (100 mg twice a day) after a meal. Following the increase in drug resistance, the eradication rate of conventional therapy was decreased. In the present study, the eradication rate was only 80% for the control group. The eradication rate of the control group was slightly higher than the result obtained by Murakami et al.^33^ This is possibly attributed to the number of days of treatment being 14 instead of 7. Furthermore, bismuth was added to the control group.

Vonoprazan is a new proton pump inhibitor, which provides sustained acid inhibitory effect, and is approved for the treatment of gastro-esophageal reflux disease. During the process of the eradication of *H. pylori*, the pH level in the stomach has to be increased to fully exert the effects of the antibiotics. *H. pylori* is more susceptible to antimicrobial agents when it restores its replicative capability at a pH level higher than 6.^12^ In the present study, the *H. pylori* eradication efficacy of vonoprazan and esomeprazole was compared. Vonoprazan triple therapy and vonoprazan quadruple therapy could significantly increase the eradication rate of *H. pylori*, suggesting that the increase in the pH levels in the stomach by using vonoprazan could achieve optimal eradication rate.

The recommended antibiotics for the eradication of *H. pylori* in the clinic include the following: amoxicillin, furazolidone, clarithromycin, metronidazole, and levofloxacin.^34^ Since the resistance rate of clarithromycin, metronidazole, and levofloxacin is very high, these drugs are used less frequently, whereas amoxicillin and furazolidone are used more frequently. Certain side effects, such as nausea, vomiting, diarrhea, and dizziness, are usual, and consequently, many patients quit the treatment schedule. The side effects are related to the duration of eradication of *H. pylori* using furazolidone as the main antibiotic treatment. In the present study, the side effects of vonoprazan triple therapy and vonoprazan quadruple therapy were examined. The former exhibited reduced side effects than the latter, whereas it had almost the same eradication rate. Therefore, it would be better to select vonoprazan plus bismuth triple therapy as the priority choice for the eradication of *H. pylori*.

There were some limitations concerning this study. First, this was a single-center study, and there were only 120 patients recruited for study; it would be better if we conducted multi-center study and recruited more patients, and the results would be more accurate. Second, in our study, patients were treated for 14 days, and we only analyzed the results of patients treated for 14 days; it would be better if we added more groups with patients treated for 7 and 10 days.

In conclusion, the present study indicated that the eradication of *H. pylori*. was decreasing, whereas the adverse effect was increasing. Both vonoprazan triple therapy and vonoprazan quadruple therapy could increase the eradication rate of *H. pylori*. Vonoprazan triple therapy exhibited reduced side effects and can be applied in the eradication of *H. pylori* in the clinic.

## Figures and Tables

**Table 1.** Baseline Characteristics of 3 Groups of Patients

General Information	Traditional Quadruple Therapy Group	Vonoprazan Triple Therapy Group	Vonoprazan Quadruple Therapy Group
Male	25	23	22
Female	15	17	18
Age (year)	50.82 ± 4.83	52.14 ± 5.37	51.36 ± 3.94
Height (cm)	171 ± 5.89	170 ± 6.73	172 ± 7.68
Body mass index (kg/m^2^)	23.2 ± 4.1	22.8 ± 2.6	23.5 ± 3.5

**Table 2.** Comparison of *H. pylori* Eradication Rate of the 3 Groups

	*H. pylori-*Negative Cases	*H. pylori*-Positive Cases	*H. pylori* Eradication Rate (%)
Traditional quadruple therapy group	32	8	80
Vonoprazan triple therapy group	38	2	95
Vonoprazan quadruple therapy group	39	1	97.5

**Table 3.** Comparison of Adverse Effects of the 3 Groups

	Traditional Quadruple Therapy Group	Vonoprazan Triple Therapy Group	Vonoprazan Quadruple Therapy Group
Nausea	3	2	1
Vomiting	1	0	1
Diarrhea	1	0	1
Abdominal distention	4	1	2
Constipation	1	0	1
Adverse effect rate (%)	25	7.5	15
